# Data on microplastics in the digestive tracts of 19 fish species from the Yellow Sea, China

**DOI:** 10.1016/j.dib.2019.103989

**Published:** 2019-05-12

**Authors:** Yongfang Zhao, Xiaoxia Sun, Qingjie Li, Yongqiang Shi, Shan Zheng, Junhua Liang, Tao Liu, Ziyang Tian

**Affiliations:** a Jiaozhou Bay National Marine Ecosystem Research Station, Institute of Oceanology, Chinese Academy of Sciences, Qingdao, 266071, China; b Laboratory for Marine Ecology and Environmental Science, Qingdao National Laboratory for Marine Science and Technology, Qingdao 266071, China; c University of Chinese Academy of Sciences, Beijing, 100049, China; d Yellow Sea Fisheries Research Institute, Chinese Academy of Fishery Sciences, Qingdao, 266071, China; e Center for Ocean Mega-Science, Chinese Academy of Sciences, Qingdao 266071, China

**Keywords:** Microplastics, Digestive tracts, Fish, The Yellow Sea

## Abstract

Microplastics (MPs) are the predominant form of marine plastic debris, and small enough to be ingested by marine organisms. Fish inhabiting coastal environments are susceptible to the ingestion of MPs. Presented data include the information of MPs level in the digestive tracts of 19 fish species which were caught from the Yellow Sea (31°28′52.380"∼38°49′15.540″ N, 120°42′36.840"∼124°49′06.180″E). For discussion and interpretation of the presented data, refer to the research article entitled “Characteristics and retention of microplastics in the digestive tracts of fish from the Yellow Sea” [1].

**Table:** Specifications Table

Subject area	Environmental pollution and biology
More specific subject area	MPs in fish
Type of data	Table, graph, Excel files
How data was acquired	Stereomicroscope (Stemi SV11, ZEISS, Shanghai, China), AxioCam HRc (Zeiss) and ImageJ software
Data format	Raw, Analyzed data
Experimental factors	Digestion, count and measurement, Statistics and mapping with Excel 2013 and ArcGIS 10.0.
Experimental features	Levels of microplastics in the digestive tracts of 19 fish species were determined.
Data source location	The Yellow Sea, China, Coordinates range: 31°28′52.380"∼38°49′15.540″ N, 120°42′36.840"∼124°49′06.180″E.
Data accessibility	The data are available in this article.
Related research article	X. Sun, Q. Li, Y. Shi, Y. Zhao, S. Zhen, J. Liang, T. Liu, Z. Tian, Characteristics and retention of microplastics in the digestive tracts of fish from the Yellow Sea, Environ. Pollut., 249, 2019, 878–885 [1].

**Table:** 

**Value of the data** • The data could be potentially valuable to the scientific community because it is new data about microplastics in fish which were caught from coastal waters. • Data are geo-referenced and it can be used in ecological risk assessment modeling. • The data has an important role in the study of China's offshore fisheries because 19 species of fish are the dominant species in the Yellow Sea.

## 1 Data

**Fig. 1:**
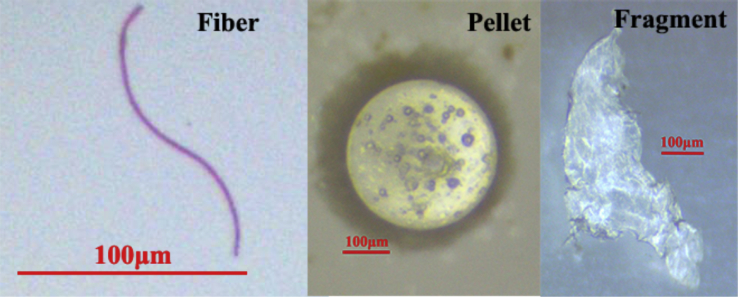
Picture of three forms of microplastics MPs.

**Fig. 2:**
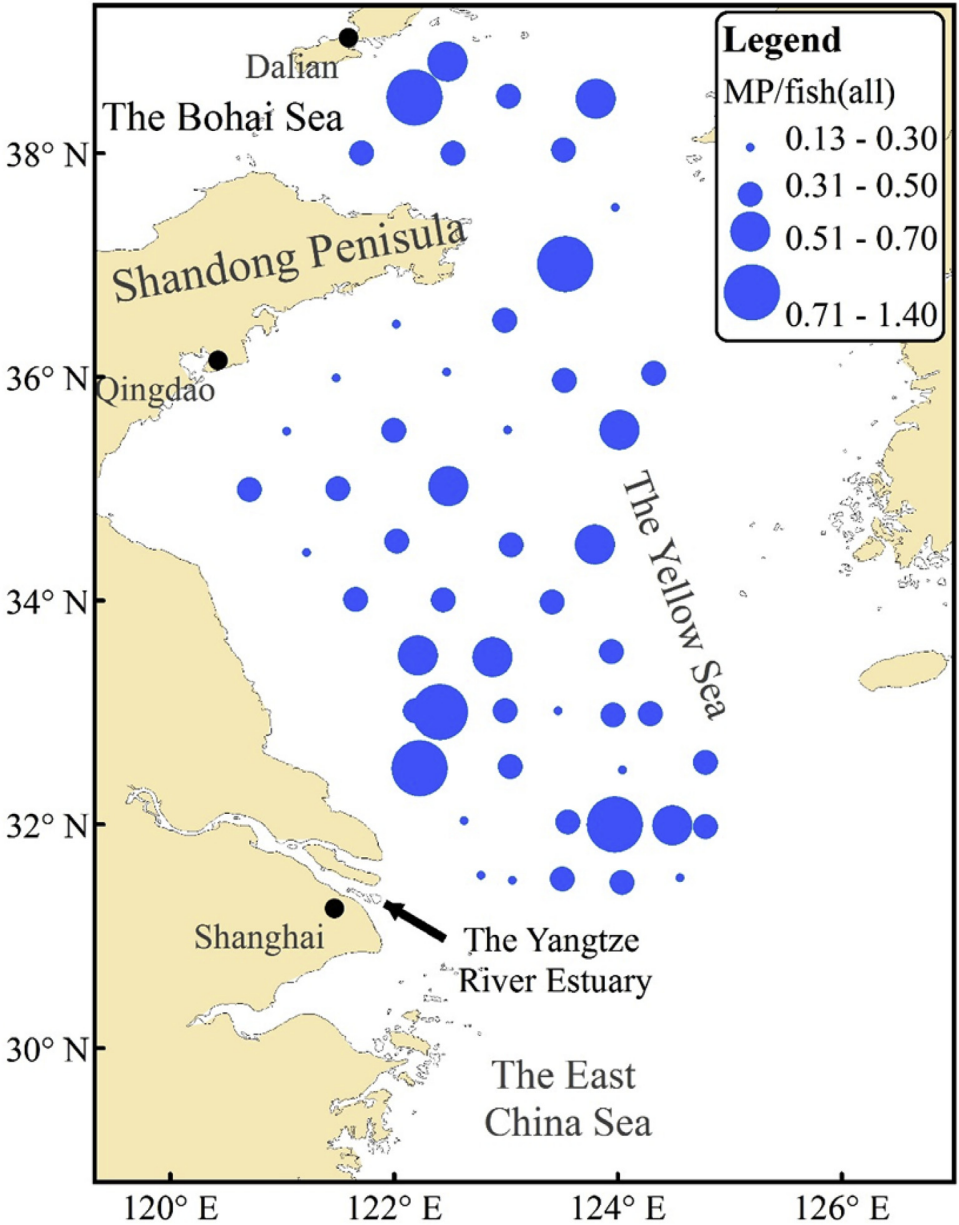
Spatial distribution of MP/fish (all).

**Fig. 3:**
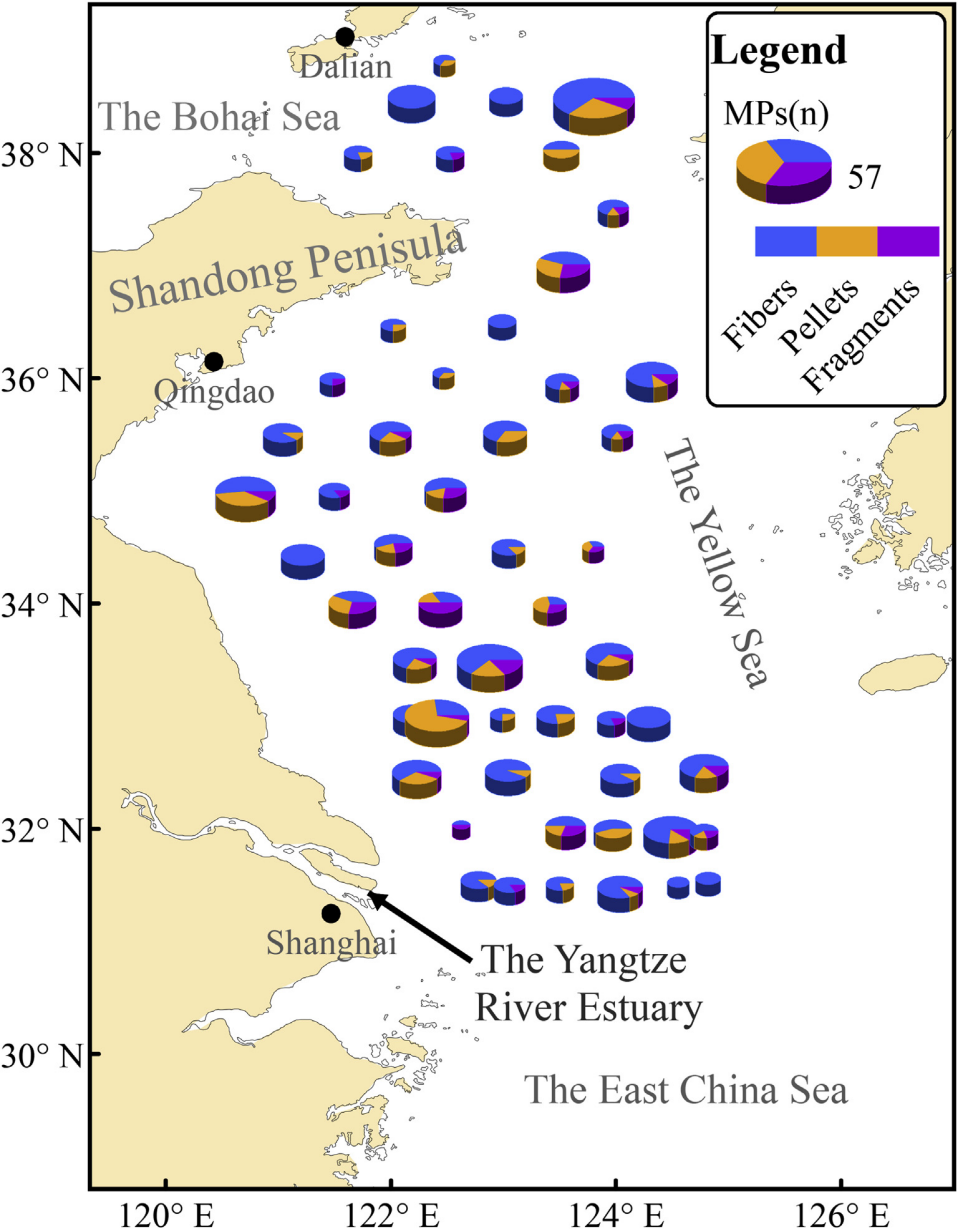
Spatial distribution of the number of MPs and the percentages of the 3 shapes of MPs.

The GPS data of 53 sampling stations and information on where 19 species of fish were sampled during June 15–30, 2016 within the Yellow Sea are shown in Table 1. Data on shapes and length of MPs are shown in Supplementary Material Table S, including the length and weight of MP source fish. Ten parameters for each station are shown in Table 2. The pictures of 3 forms of MPs are shown in Fig. 1. The spatial distribution of MP/fish(all) is shown in Fig. 2. A comparison of the spatial distribution of fibers and other MPs is shown in Fig. 3. The spatial patterns can be used for comparison studies in the future by choosing similar monitoring programs.

**Table 1:** Fish species and GPS data of the sampling stations.

Station	Latitude	Longitude	Fish species: Number of fish (n)				Total number of fish(n)
A01	38°49′15.540″	122°28′37.080″	⑥5				5
A02	38°29′56.400″	122°11′07.140″	⑲10				10
A03	38°30′32.340″	123°01′27.300″	⑲20				20
A04	38°29′14.760″	123°48′12.420″	④20	⑥20	⑫15	⑲20	75
A05	38°00′12.720″	121°42′36.420″	⑥10				10
A06	37°59′59.520″	122°31′35.100″	⑰10				10
A07	38°01′50.460″	123°30′54.600″	⑥10	⑨6			16
A08	37°30′57.300″	123°58′39.060″	⑥20				20
A09	37°00′30.600″	123°31′54.000″	⑥10	⑫10			20
A10	36°28′20.580″	122°01′22.020″	②5	⑥5	⑮10		20
A11	36°30′24.540″	122°59′16.380″	⑥10				10
A12	35°59′37.320″	121°28′56.700″	⑯20				20
A13	36°02′46.380″	122°28′09.480″	⑥10				10
A14	35°58′13.680″	123°31′21.120″	⑩10	⑫10			20
A15	36°01′56.940″	124°19′07.020″	⑥20	⑨20			40
A16	35°30′51.000″	121°02′34.260″	⑦20	⑫10	⑮10	⑰10	50
A17	35°31′20.340″	121°59′58.800″	⑥10	⑧20			30
A18	35°31′41.340″	123°00′52.500″	⑧20	⑫9	⑮15		44
A19	35°31′31.440″	124°00′44.400″	⑥5	⑮5			10
A20	34°59′45.000″	120°42′36.840″	⑥10	⑦20	⑩10	⑫20	60
A21	35°00′08.880″	121°30′02.340″	⑫5	⑮10			15
A22	35°01′23.760″	122°29′13.920″	⑨10	⑮10			20
A23	34°25′51.960″	121°13′12.060″	⑩20	⑪20	⑬10		50
A24	34°31′53.340″	122°01′24.780″	⑥10	⑩10			20
A25	34°29′54.600″	123°02′42.180″	⑥10	⑮10			20
A26	34°30′10.200″	123°47′45.840″	⑥5				5
A27	34°00′32.400″	121°39′35.820″	⑮20	⑱20			40
A28	34°00′19.680″	122°26′23.760″	⑥6	⑮20			26
A29	33°59′15.540″	123°24′46.860″	①10	⑥9			19
A30	33°30′39.600″	122°12′46.920″	⑩10	⑮10			20
A31	33°29′44.040″	122°52′45.780″	⑩20	⑮30			50
A32	33°32′48.414″	123°56′32.820″	①10	⑥10	⑩20		40
A33	33°01′00.774″	122°11′23.940″	③10	⑮10			20
A34	33°00′19.920″	122°24′42.840″	③10	⑭10	⑮10		30
A35	33°00′55.674″	122°59′34.860″	③10				10
A36	33°00′55.380″	123°27′50.400″	⑥10	⑭20			30
A37	32°58′48.360″	123°57′31.380″	⑮10				10
A38	32°59′25.560″	124°17′20.820″	⑩20	⑮10			30
A39	32°30′06.714″	122°13′51.240″	⑮10	⑱10			20
A40	32°31′04.500″	123°02′23.280″	②10	⑮20	⑯8		38
A41	32°29′13.794″	124°02′24.000″	⑥10	⑩17	⑭10		37
A42	32°33′13.200″	124°46′55.200″	⑥10	⑩10	⑭10	⑮10	40
A43	32°01′58.320″	122°37′30.780″	⑮10				10
A44	32°01′17.574″	123°33′09.720″	⑭10	⑮10			20
A45	31°59′53.760″	123°58′14.520″	⑩10				10
A46	31°59′24.300″	124°29′09.000″	⑭10	⑯20			30
A47	31°58′42.480″	124°46′54.780″	⑮10				10
A48	31°32′36.660″	122°46′43.260″	⑮20	⑯20			40
A49	31°30′00.180″	123°03′20.280″	⑤10	⑯10			20
A50	31°30′35.340″	123°30′05.640″	⑭10				10
A51	31°28′52.380″	124°02′14.160″	⑩10	⑭20			30
A52	31°31′27.180″	124°33′16.140″	⑥10	⑭10			20
A53	31°33′26.160″	124°49′06.180″	⑥10	⑩10	⑭10		30

① *Lophius litulon*

② *Eupleurogramms muticus*

③ *Larimichthys polyactis*

④ *Enchelyopus elongatus*

⑤ *Psenopsis anomala*

⑥ *Liparis tanakae*

⑦ *Hexagrammos otakii*

⑧ *Gadus macrocephalus*

⑨ *Cleisthenes herzensteini*

⑩ *Chelidonichthys kumu*

⑪ *Setipinna taty*

⑫ *Pholis fangi*

⑬ *Pampus argenteus*

⑭ *Erisphex pottii*

⑮ *Engraulis japonicus*

⑯ *Decapterus maruadsi*

⑰ *Apogon lineatus*

⑱ *Anchoviella commersonii*

⑲ *Ammodytes personatus*

**Table 2:** Statistical data based on different stations.

Station	The total number of fish (n)	The mean of fish length(with MP) (cm)	The mean of fish weight(with MP) (g)	The total number of fiber MPs (n)	The total number of pellet MPs (n)	The total number of fragment MPs (n)	The mean of MPs length(μm)	IR	MP/fish (all)	MP/fish (with MP)
A01	5	8.96	8.13	2	1	0	1109	0.40	0.60	1.5
A02	10	8.88	2.09	14	0	0	960	0.80	1.40	1.8
A03	20	9.47	3.09	7	0	0	923	0.30	0.35	1.2
A04	75	13.64	12.12	26	13	3	881	0.48	0.56	1.2
A05	10	8.27	8.11	4	1	0	1457	0.40	0.50	1.3
A06	10	6.46	3.46	4	0	1	967	0.40	0.50	1.3
A07	16	9.27	11.54	4	4	0	780	0.44	0.50	1.1
A08	20	8.34	6.15	4	1	1	802	0.25	0.30	1.2
A09	20	11.50	8.98	8	5	5	717	0.60	0.90	1.5
A10	20	21.34	12.45	3	1	0	1943	0.20	0.20	1.0
A11	10	8.32	5.69	5	0	0	1694	0.50	0.50	1.0
A12	20	10.91	13.07	3	0	1	905	0.20	0.20	1.0
A13	10	9.21	9.00	2	1	0	1164	0.30	0.30	1.0
A14	20	15.81	23.68	5	1	1	784	0.30	0.35	1.2
A15	40	8.65	6.51	13	2	2	876	0.35	0.43	1.2
A16	50	10.13	13.53	9	1	0	1548	0.20	0.20	1.0
A17	30	8.96	5.88	7	3	1	1220	0.33	0.37	1.1
A18	44	9.96	8.56	8	4	0	869	0.27	0.27	1.0
A19	10	12.00	18.93	4	1	1	1106	0.50	0.60	1.2
A20	60	10.02	8.96	12	9	2	877	0.33	0.38	1.2
A21	15	11.17	8.86	5	0	1	863	0.33	0.40	1.2
A22	20	13.85	20.00	6	2	3	614	0.45	0.55	1.2
A23	50	13.18	18.05	12	0	0	917	0.22	0.24	1.1
A24	20	10.47	12.36	5	2	2	979	0.40	0.45	1.1
A25	20	12.71	17.40	6	1	0	1086	0.35	0.35	1.0
A26	5	8.46	6.80	1	1	1	495	0.40	0.60	1.5
A27	40	7.74	2.92	6	4	4	662	0.30	0.35	1.2
A28	26	10.41	10.34	4	2	6	1110	0.38	0.46	1.2
A29	19	13.26	41.40	2	3	2	609	0.37	0.37	1.0
A30	20	8.78	6.30	8	3	1	1528	0.55	0.60	1.1
A31	50	9.74	7.85	17	6	4	707	0.40	0.54	1.4
A32	40	11.55	25.16	9	4	1	950	0.28	0.35	1.3
A33	20	7.54	2.80	7	1	1	1382	0.35	0.45	1.3
A34	30	7.25	3.76	7	18	1	168	0.33	0.87	2.6
A35	10	7.60	3.47	3	1	0	335	0.30	0.40	1.3
A36	30	8.22	7.78	7	2	0	562	0.27	0.30	1.1
A37	10	9.35	4.79	4	0	1	841	0.40	0.50	1.3
A38	30	11.26	13.25	12	0	0	1242	0.40	0.40	1.0
A39	20	7.57	2.65	9	5	1	647	0.60	0.75	1.3
A40	38	15.72	9.36	12	1	0	1660	0.26	0.34	1.3
A41	37	9.80	15.34	9	1	0	1270	0.22	0.27	1.3
A42	40	11.14	14.84	10	3	2	1154	0.30	0.38	1.3
A43	10	9.24	5.58	1	0	1	273	0.20	0.20	1.0
A44	20	9.62	8.78	5	2	3	690	0.40	0.50	1.3
A45	10	12.16	18.06	5	4	0	919	0.60	0.90	1.5
A46	30	9.57	11.05	14	3	2	1220	0.43	0.63	1.5
A47	10	11.57	11.83	3	1	1	729	0.50	0.50	1.0
A48	40	8.74	6.11	7	1	0	1405	0.18	0.20	1.1
A49	20	10.76	15.00	5	0	1	801	0.25	0.30	1.2
A50	10	8.02	10.02	4	1	0	756	0.40	0.50	1.3
A51	30	9.67	13.28	11	1	1	1162	0.37	0.43	1.2
A52	20	10.09	17.65	3	0	0	588	0.15	0.15	1.0
A53	30	10.91	17.22	4	0	0	1568	0.13	0.13	1.0

## 2 Experimental design, materials, and methods

### 2.1 Sample collections

Fish were collected from each site using a bottom trawling net. The perimeter of the mouth of the trawling net was 167.2 m and the net was 83.2 m long, with a mesh size of 2.4 cm. The net was trawled behind the ship for 1 h at each station at a speed of 3 knots. All of the fish collected during the trawl were removed from the net and frozen immediately.

### 2.2 Fish species identifications and biological measurements

For each site, only the species which number was more than 5 fishes were selected for the next analysis. After measuring the body length and weight of each fish, the digestive tract was removed, placed in a 20-mL scintillation vial and fixed in formaldehyde solution. In total, 1320 fish belonging to 19 species were studied.

### 2.3 Digestion and measurement of MPs from the digestive tracts of fish

The digestive tract was removed from every fish, placed in a 20-mL scintillation vial and fixed in formaldehyde solution. The HNO3 solution digestion method described by Desforgeset al. (2015) [2] was used to destroy the digestive tract of fish. Briefly, 100% HNO3 was added to each vial, after which the vials were covered and heated in a water bath at approximately 80 °C for 3 h until the digestive tract was completely digested. The digested samples were then passed through 0.45-μm mixed cellulose ester filter papers, and the filter papers were subsequently checked for MPs under a stereomicroscope (Stemi SV11, ZEISS, Shanghai, China). In addition, three blanks with only HNO3 were run for each batch of samples to correct for potential air-borne MPs deposition in the laboratory. No contamination occurred during the experiment.

All of the MPs detected in fish samples were counted and imaged with an AxioCam HRc (Zeiss) connected to a stereomicroscope (Stemi SV11, ZEISS, Shanghai, China). The length of each MP was measured manually using the ImageJ software. All of the MPs detected in fish were classified into three categories: fibers, pellets, and fragments.

### 2.4 Data processing

Three parameters were calculated, the incidence rate (IR), MP/fish(all), MP/fish(with MP).

$$\text{IR}=\;\frac{\text{FishMN}}{\text{FishN}}$$

$$\text{MP}/\text{fish}\;\left(\text{all}\right)=\frac{\text{MPsN}}{\text{FishN}}$$

$$\text{MP}/\text{fish}\;\left(\text{with}\;\text{MP}\right)=\frac{\text{MPsN}}{\text{FishMN}}$$

MPsN: the number of MPs.
FishN: the total number of fish.
FishMN: the number of fish contained MPs.
Plots were created using ArcGIS 10.0 and Microsoft Excel 2013.
