# Supplementary material for: Data on microplastics in the digestive tracts of 19 fish species from the Yellow Sea, China
Source: Data Brief. 2019 May 12;25:103989. doi: 10.1016/j.dib.2019.103989 (PMC6626890; doi:10.1016/j.dib.2019.103989)
Supplement: Supplementary file 1 — Multimedia component 1 [file mmc1.pdf]

Author's agreement

We the undersigned declare that the manuscript entitled "**Data on microplastics in the digestive tracts of 19 fish species from the Yellow Sea, China**" is original, has not been full or partly published before, and is not currently being considered for publication elsewhere.

We confirm that the manuscript has been read and approved by all named authors and that there are no other persons who satisfied the criteria for authorship but are not listed. We further confirm that the order of authors listed in the manuscript has been approved by the undersigned.

We understand that the Corresponding Author is the sole contact for the editorial process The corresponding author "Xiaoxia Sun" is responsible for communicating with the other authors about progress, submissions of revisions and final approval of proofs.

Signature of all authors: Xiaoxia Sun

Yongfang Zhao

Junhua Liang

Yongqiang Shi

Tao Liu

Ziyang Tian

Shan Zheng
